# Catalytic mechanism of ancestral L-lysine oxidase assigned by sequence data mining

**DOI:** 10.1016/j.jbc.2021.101043

**Published:** 2021-08-04

**Authors:** Sayaka Sugiura, Shogo Nakano, Masazumi Niwa, Fumihito Hasebe, Daisuke Matsui, Sohei Ito

**Affiliations:** 1 Graduate Division of Nutritional and Environmental Sciences, University of Shizuoka, Suruga-ku, Shizuoka, Japan; 2 PREST, Japan Science and Technology Agency, Kawaguchi, Japan; 3 Department of Biotechnology, College of Life Sciences, Ritsumeikan University, Kusatsu, Shiga, Japan

**Keywords:** L-amino acid oxidase, crystal structure, ancestral sequence reconstruction, enzymatic mechanism, 5-APNA, 5-aminopentanoic acid, 5-APNM, 5-aminopentanamide, AncLLysO(LF), ligand-free form of AncLLysO, AncLLysO, ancestral LLysO, AROD, L-arginine oxidase, ASR, ancestral sequence reconstruction, FAO, flavin-dependent amine oxidase, L-LOX/MOG, L-amino acid oxidase/monooxygenase, LAAO, L-amino acid oxidase, LLysO, L-lysine α-oxidase

## Abstract

A large number of protein sequences are registered in public databases such as PubMed. Functionally uncharacterized enzymes are included in these databases, some of which likely have potential for industrial applications. However, assignment of the enzymes remained difficult tasks for now. In this study, we assigned a total of 28 original sequences to uncharacterized enzymes in the FAD-dependent oxidase family expressed in some species of bacteria including *Chryseobacterium*, *Flavobacterium*, and *Pedobactor*. Progenitor sequence of the assigned 28 sequences was generated by ancestral sequence reconstruction, and the generated sequence exhibited L-lysine oxidase activity; thus, we named the enzyme AncLLysO. Crystal structures of ligand-free and ligand-bound forms of AncLLysO were determined, indicating that the enzyme recognizes L-Lys by hydrogen bond formation with R76 and E383. The binding of L-Lys to AncLLysO induced dynamic structural change at a plug loop formed by residues 251 to 254. Biochemical assays of AncLLysO variants revealed the functional importance of these substrate recognition residues and the plug loop. R76A and E383D variants were also observed to lose their activity, and the *k*_cat_/*K*_m_ value of G251P and Y253A mutations were approximately 800- to 1800-fold lower than that of AncLLysO, despite the indirect interaction of the substrates with the mutated residues. Taken together, our data demonstrate that combinational approaches to sequence classification from database and ancestral sequence reconstruction may be effective not only to find new enzymes using databases of unknown sequences but also to elucidate their functions.

Amino acids are key biomolecules that regulate physiological processes. Proteins and many bioactive compounds are synthesized utilizing L-amino acids and their metabolites, whereas D-amino acids serve as precursors of peptide drugs. Many L-amino acid–metabolizing enzymes have been reported, and several of them are used to synthesize fine chemicals (1), such as amino acid racemase (2) and L-amino acid dehydrogenase (3). Among these enzymes, L-amino acid oxidase (LAAO) is expected to have many industrial and pharmaceutical applications (4, 5). LAAOs are FAD-dependent enzymes and are broadly expressed in many species, from bacteria to mammals (6, 7, 8, 9, 10). LAAO catalyzes the oxidation of the main chain amino group of L-amino acids and produces imino acids; these products are released into solvents and are quickly hydrolyzed to keto acid (4). The reduced FAD is reoxidized by oxygen molecules and generates H_2_O_2_ as a by-product (4). LAAO activity can be estimated by quantifying H_2_O_2_ using the Trinder reaction.

LAAOs have broad substrate selectivity, which enables their varied applications. For example, the LAAOs from *Rhodococcus opacus* (8) and *Proteus myxofaciens* (11) can be utilized to deracemize racemic amino acids to D-amino acids as efficiently as L-amino acid deaminase (12, 13, 14, 15). On the other hand, LAAOs bearing high substrate specificity could be used to quantify specific L-amino acid concentrations in various samples. These LAAOs are named based on their substrate specificity and are the main focus of this study. Examples of these LAAOs include L-aspartate oxidase (16, 17) and L-glutamate oxidase (18, 19).

The assignment of new LAAOs aids in advancing the application of these enzymes. In addition, structural and functional analyses of LAAOs would be helpful to predict how LAAOs acquired unique substrate selectivity toward different L-amino acids through the molecular evolutionary process. Currently, many research groups have succeeded in experimentally screening LAAOs from various species (4, 5, 9). Simultaneously, the recent expansion of registered protein sequence data in public databases enables us to find new LAAOs using *in silico* enzyme screening methods and ancestral sequence reconstruction (ASR). Here, ASR is a sequence-based protein redesign method that can generate ancestral proteins located at each node of the phylogenetic tree (20, 21). Ancestral proteins often have desirable properties for use in practical applications, such as high thermostability (22, 23) and broad substrate selectivity (24). Therefore, ASR is currently being adopted as a tool for protein engineering (25). For example, new LAAOs that bear broad substrate selectivity (>10 L-amino acids) could be assigned by a paralog search of L-arginine oxidase (AROD) (26, 27, 28). Artificial LAAOs can be designed by ASR utilizing six of the assigned LAAOs as a sequence library. The designed enzyme, called AncLAAO, could be produced using the *Escherichia coli* expression system with the highest yield among any previously reported LAAOs (>50 mg/l). These can then be applied to deracemize dozens of racemic amino acid derivatives to their D-forms with high enantiopurity (26). On the other hand, there are thousands of protein sequences belonging to the LAAO superfamily in databases (29), and the currently available functionally characterized sequences are only the tip of the iceberg. Several sequences, such as AROD (27, 30), AncLAAO (26, 28), and L-tryptophan oxidase (VioA) (31, 32, 33), can now be functionally annotated. Thus, there is an opportunity to acquire new LAAOs from the database using *in silico* enzyme screening methods.

In this study, we attempted to assign new LAAOs from the database utilizing one of the previously designed AncLAAO sequences (28) as a template. Through combinational approaches using paralog searches, the application of previously reported original protein sequence selection methods (27, 34, 35, 36), and biochemical assays, we newly characterized LAAOs exhibiting high specificity toward L-Lys. These included L-Lysine α-oxidase (LLysO) from the *Chryseobacterium*, *Flavobacterium*, and *Pedobacter* species. The assigned LLysO has low sequence identity (less than 30%) to the previously reported LLysO from *Trichoderma viridae* (TvLysOX) (37, 38) and L-amino acid oxidase/monooxygenase (L-LOX/MOG) from *Pseudomonas* sp. AIU813 (39, 40, 41); therefore, the enzymatic properties of the assigned LLysO could not be estimated only from the sequence analysis. Ancestral LLysO (AncLLysO) was designed utilizing the assigned LLysO sequences as templates. Compared with native LLysO, AncLLysO improved thermal stability and provided good quality of crystals. The combination of biochemical and structural analysis of AncLLysO and its variants revealed the substrate recognition and reaction mechanism of LLysO at a molecular level.

## Results

### Sequence classification from databases to assign a new LLysO family

**Figure 1:**
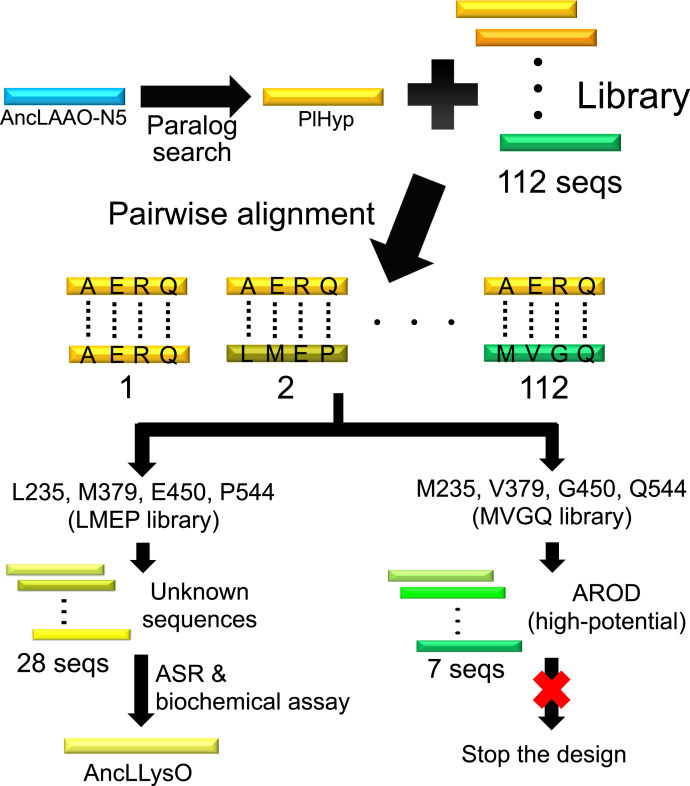
**Schematic view indicating how to design AncLLysO from AncLAAO-N5 sequence data.** Paralog sequence of L-amino acid oxidase (LAAO), named PlHyp (GI: WP_063360054.1), was assigned from *Pseudoalteromonas luteoviolacea*, similar to the assignment of AncLAAO (26). A library containing a total 112 homolog sequences to PlHyp was prepared by applying Blastp analysis and the preprocessing approach. The library was classified into two curated libraries using four key residues (235, 379, 450, and 544) as sequence motifs and pairwise alignment. The key residues were assigned by applying the same procedure used in previous studies (35, 36). Sequences bearing M235, V379, G450, and Q544 (*green bar*, total seven sequences) exhibit L-arginine oxidase (AROD) activity because they share high sequence identity with the enzyme; thus, we halted further design. On the other hand, sequences bearing L235, M379, E450, and P544 (*yellow bar*, total 28 sequences) had no sequence identity to already reported enzymes. The ancestral protein of the curated sequences was generated by ancestral sequence reconstruction (ASR) the designed sequence was named AncLLysO based on the enzymatic activity.

In a previous study, we reported that paralog search and sequence library classification using several key residues as a motif are effective for finding new enzymes from sequence databases bearing unique properties, such as broad substrate selectivity and high thermostability (26, 35, 36). By applying combinational approaches (represented in Fig. 1), we attempted to assign new LAAOs from the databases using the following procedures. First, paralogs that bear moderate sequence identity (<30%) with AncLAAO-N5 (28) were identified by Blastp utilizing open reading frames of six *Pseudoalteromonas* genera as a library. As shown in a previous study, these genera have LAAO sequences that were utilized to design AncLAAO-N5 (26, 28). Through this analysis, a paralog sequence was assigned in *Pseudoalteromonas luteoviolacea*; the sequence identity was quite low compared with the functionally annotated LAAOs. This paralog sequence was called PIHyp (Fig. 1).

Next, we attempted to classify homologous sequences of PIHyp registered in the sequence database by applying the previously reported approach (35, 36). To achieve this, the sequences were prepared by submitting PIHyp to Blastp (42); the database and E-values were set to “nonredundant” and 1.0∗10^−6^, respectively. Finally, a total of 112 sequences could be obtained by eliminating sequences that were more than 5% longer or shorter than PIHyp and that shared a greater than 90% identity with other sequences; these sequences were utilized as the library (Fig. 1). The elimination was performed using an original python script (35). We attempted to assign key residues to curate the library by following previously reported procedures (35, 36). Four residues (the 235th, 379th, 450th, and 544th residues) were assigned as key residues by the analysis. After pairwise alignment between PIHyp and each of the sequences in the library (total 1–112 sequences), the sequences were classified into two groups based on the combination of the key residues: one had Leu, Met, Glu, and Pro (LMEP, yellow bar in Fig. 1) and the other had Met, Val, Gly, and Gln (MVGQ, green bar in Fig. 1). Finally, a total of 28 LMEP and seven MVGQ sequences were acquired (Fig. 1).

The quality of the sequence libraries could be evaluated by comparing conservation energies before and after the classification (34, 36). Conservation energies at the *i*^th^ residues (*E*_*aa,i*_) and the sum of all residues (*E*_*c*_) could be calculated with the previously reported equation:

$$E_{aa,i}=-\ln\;f_{aa,i},E_{c}=\sum_{i=1}^{n}E_{aa,i}$$

where *f*_*aa,i*_ is the frequency at the *i*^th^ positions of the target protein (PIHyp, in this case) in the alignment, and *n* is the total number of residues in the target protein (43). The *E*_*c*_ value would be close to zero if the curation is correctly performed (36). The distribution of the *E*_*aa,i*_ value for before and after the classification is represented in Fig. S1. The *E*_*c*_ value for the LMEP (102.6 in Fig. S1*B*) and MVGQ (129.5 in Fig. S1*C*) library was more than 3-fold lower than that of the noncurated library (397.9 in Fig. S1*A*), suggesting that the library could be classified to align the sequences accurately. Clearly assigned consensus residues (residues 35–44, 269–278, and 316–323) after curation also supported this point (Fig. S1).

Here, the seven MVGQ sequences would have L-arginine oxidase (AROD) activity because they shared more than 70% identity with the already reported AROD sequences, and therefore, we discontinued further design. On the other hand, the 28 LMEP sequences share less than 30% identity with the already characterized LAAOs, suggesting that the sequences had novel activities. Preliminary enzyme activity assay for one of the sequences from *Chryseobacterium angstadtii* (CaLLysO, WP_048506397.1, Table S1) suggested that the sequences would have L-Lys oxidase (LLysO) activity. To prove this point, the ancestral protein of the 28 LMEP sequences, named AncLLysO (Table S2), was designed as a representative of the curated sequences; AncLLysO would have LLysO activity if the 28 LMEP sequences bear this activity.

### Enzyme functional analysis of CaLLysO and AncLLysO

**Figure 2:**
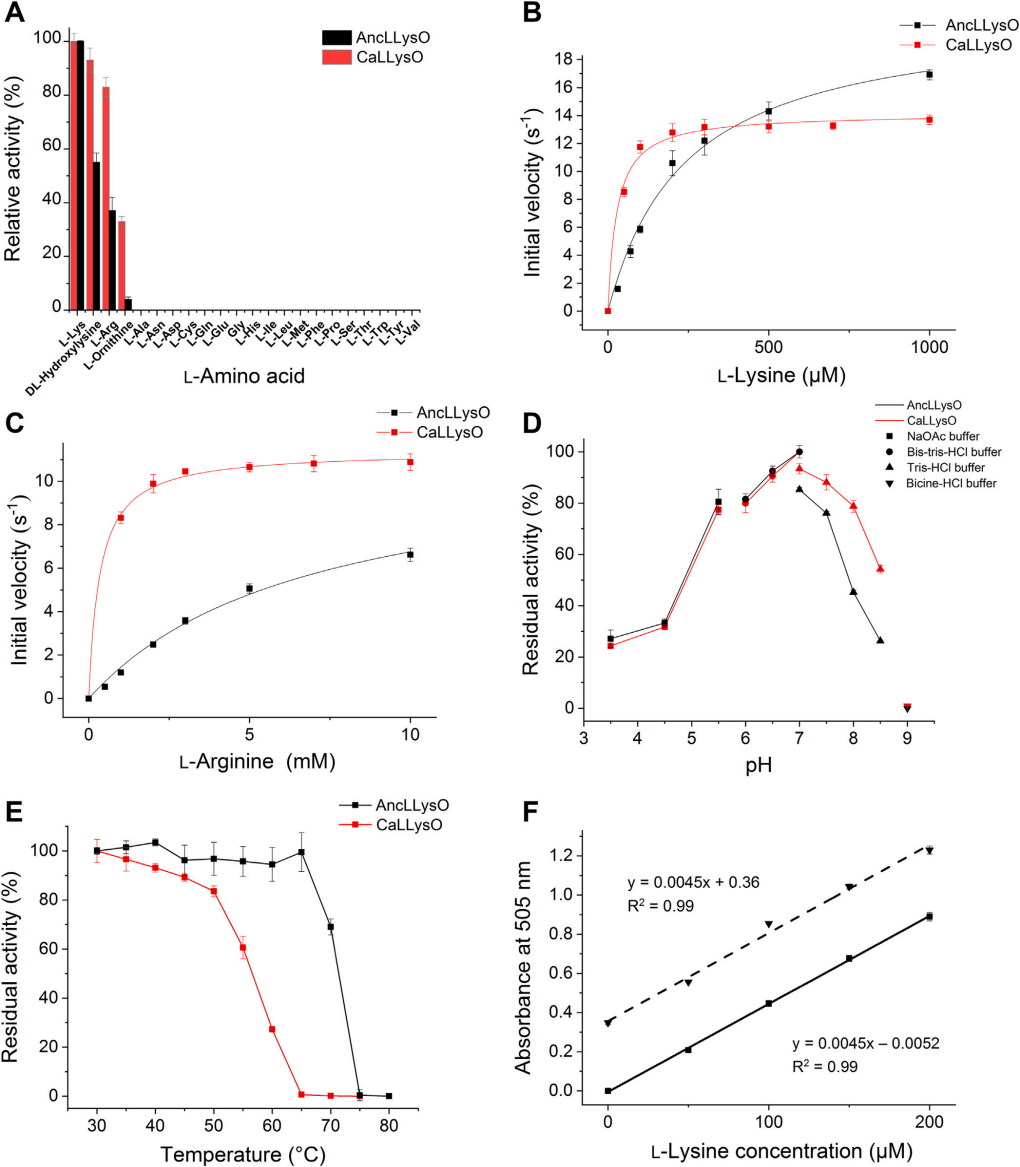
**Biochemical analysis of CaLLysO and AncLLysO.** Relative specific activity of CaLLysO and AncLLysO toward different amino acid substrates (10 mM) (*A*). The parameters of CaLLysO and AncLLysO are represented as *red* and *black* in the figure. Both enzymes exhibited the highest activity toward L-Lys. Enzyme kinetic plots of CaLLysO and AncLLysO toward L-Lys (*B*) and L-Arg (*C*), respectively. Analysis of pH optima (*D*) and thermostability (*E*). Optimal pH value of CaLLysO and AncLLysO was 7.0. The *t*_1/2_ values of CaLLysO and AncLLysO were approximately 57 and 73 °C, respectively. Quantification of L-Lys concentration by AncLLysO (*F*). Samplescontaining human plasma (*downward triangle*) and only buffer (*square*) are shown as *dashed* and *straight lines*, respectively. The assay was performed by endpoint method. All of the experiments were performed using two biological replicates per three technical replicates.

Enzymatic properties of CaLLysO and AncLLysO were analyzed using biochemical assays and suggested that substrate specificity and pH dependency were similar to each other. Specific activity measurement of CaLLysO (red bar in Fig. 2*A*) and AncLLysO (black bar in Fig. 2*A*) toward 20 L-amino acids and two derivatives (L-Ornithine [L-Orn] and 5-hydroxy-DL-Lysine [5-OH-DL-Lys]) indicated that both of the LLysOs exhibited strong activity toward basic amino acids in the following order: L-Lys > 5-OH-DL-Lys > L-Arg > L-Orn (Fig. 2*A*). Enzyme kinetic analysis toward L-Lys (Fig. 2*B*) and L-Arg (Fig. 2*C*) indicated that both CaLLysO and AncLLysO had the highest enzyme efficiency (*k*_cat_/*K*_m_) values toward L-Lys compared with other substrates; the values of L-Lys were >15- and 42-fold higher than those of L-Arg in CaLLysO and AncLLysO, respectively (Table 1). The pH analysis indicated that both CaLLysO and AncLLysO exhibited the highest activity at pH 7.0 (Fig. 2*D*). On the other hand, a functional trade-off between thermostability and *k*_cat_/*K*_m_ value toward L-Lys was observed between CaLLysO and AncLLysO; the *t*_1/2_ value of AncLLysO was approximately 15 °C higher than that of CaLLysO (Fig. 2*E*), whereas the *k*_cat_/*K*_m_ value of AncLLysO was more than 7-fold lower than that of CaLLysO (Table 1). Thus, AncLLysO is more thermophilic than CaLLysO.

**Table 1:** Enzyme kinetic parameters of AncLLysO and CaLLysO toward the substrates L-Lys, L-Arg, and L-Orn

Substrate	CaLLysO			AncLLysO		
	*k*_cat_	*K*_m_	*k*_cat_/*K*_m_	*k*_cat_	*K*_m_	*k*_cat_/*K*_m_
	s^−1^	mM	s^−1^ mM^−1^	s^−1^	mM	s^−1^ mM^−1^
L-Lys	14.2 ± 0.3	0.027 ± 0.003	516	21.7 ± 0.9	0.3 ± 0.03	72.3
L-Arg	11.4 ± 0.1	0.34 ± 0.03	33.2	11.4 ± 1.0	6.9 ± 1.1	1.7
L-Orn	3.60 ± 0.07	4.4 ± 0.4	0.81	46.4 ± 3.0	45.8 ± 5.2	1.0

The measurement of enzyme kinetic parameters was performed using two biological replicates per three technical replicates.

The highly specific LLysO can be applied to quantify the concentration of L-Lys in various samples (44). Here, AncLLysO is suitable for the application because it exhibits high thermostability and a larger relative *k*_cat_/*K*_m_ value toward L-Lys in comparison with L-Arg; the *k*_cat_/*K*_m_ value was 42.5 for AncLLysO and 15.5 for CaLLysO (Table 1). Utilizing AncLLysO, the L-Lys concentration was quantified for the samples containing only buffer (straight line in Fig. 2*F*) and plasma (dotted line in Fig. 2*F*), and the results were plotted in Figure 2*F*. The slopes of the plots were identical to each other (0.045 in Fig. 2*F*), indicating that AncLLysO can quantify L-Lys concentration as well as the previously reported highly specific L-amino acid oxidases.

Summarizing the results, we showed that both CaLLysO and AncLLysO have LLysO activity. In particular, AncLLysO has favorable properties for analyzing its biochemical functions, such as high thermostability and specificity toward L-Lys. Thus, AncLLysO was adopted as a research target to reveal its enzymatic properties at a molecular level.

### LC-HRMS analysis of products generated by converting L-Lys with AncLLysO

**Figure 3:**
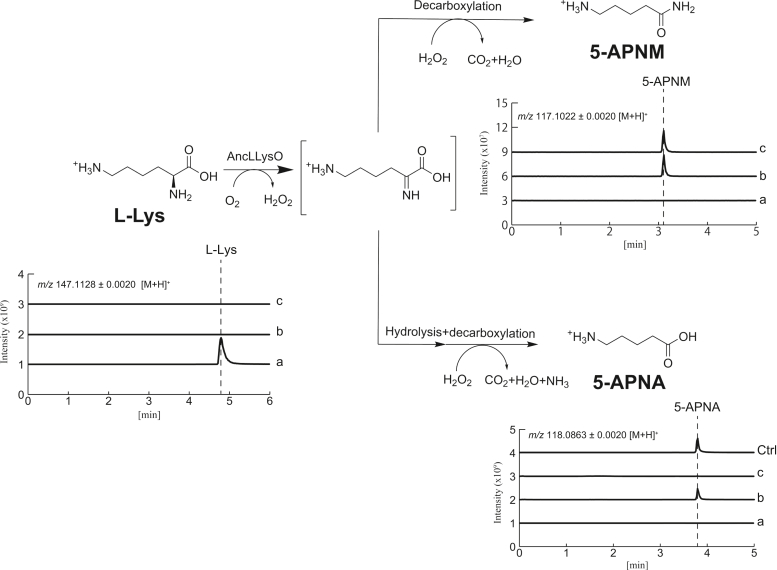
**Reaction scheme of AncLLysO predicted from LC-HRMS analysis.** Extracted ion count chromatograms of [M + H]^+^ for each compound (L-Lys, 5-APNM, and 5-APNA). Here, each chromatogram (a, b, and c) was obtained by analyzing samples after reacting for 24 h under the following conditions: a, 10 mM L-Lys; b, 10 mM L-Lys and 0.25 mg AncLLysO; and c, 10 mM L-Lys, 0.25 mg AncLLysO, and 1000 U catalase. 5-APNA, 5-aminopentanoic acid; 5-APNM, 5-aminopentanamide.

Currently, a total of two types of LLysO have been reported: one is L-Lys α-oxidase, which oxidizes the main chain amino group of L-Lys (44), and the other is L-Lys ε-oxidase, which oxidizes the side chain amino group of L-Lys (45). To ensure clarity regarding the types represented by AncLLysO identified, the reaction products of AncLLysO were analyzed by LC-HRMS (Fig. 3). Here, the reactions were conducted under the following three independent conditions: reaction buffer (100 mM KPB (7.0) and 10 mM L-Lys) containing no enzyme (a in Fig. 3), containing 0.25 mg AncLLysO (b in Fig. 3), and containing 0.25 mg AncLLysO and 1000 U catalase (c in Fig. 3). After stopping the reaction by mixing nine volumes of acetonitrile with one volume of reaction solution, the mixture was centrifuged at 20,000*g* at 4 °C for 10 min. The supernatant was analyzed by LC-HRMS. A peak corresponding with L-Lys completely disappeared following the addition of AncLLysO (b and c in Fig. 3), indicating that AncLLysO can utilize L-Lys as a substrate. The product peak analysis suggested that AncLLysO exhibits L-Lys α-oxidase activity. In fact, a peak of 5-aminopentanoic acid (5-APNA) was detected in condition “b” (Fig. 3). As shown in a previous study (41), 5-APNA was produced under the conditions containing H_2_O_2_ by hydrolyzing and decarboxylating the resultant imino acids (Fig. 3). The analysis also suggested that AncLLysO generates 5-aminopentanamide (5-APNM); the peak of 5-APNM was confirmed under conditions with (b in Fig. 3) and without catalase (c in Fig. 3), as was the case with L-LOX/MOG (41).

Referring to the analysis for L-LOX/MOG (41), we estimated the relative yield of 5-APNA and 5-APNM from their peak area value. Our analysis suggested that a larger amount of 5-APNA was produced than 5-APNM. The area value for 5-APNA was more than 15-fold larger than that of 5-APNM (b in Fig. 3), suggesting that, for AncLLysO, the monooxygenase function was the promiscuous activity compared with the oxidase function. This was clearly different in the case of L-LOX/MOG, where the monooxygenase function was the main activity identified through LC-HRMS analysis (41).

### Structural analysis of AncLLysO (ligand-free) form

As shown through biochemical analysis, AncLLysO exhibited both L-Lys α-oxidase and monooxygenase activity, and structural analysis is expected to reveal its reaction mechanism at a molecular level. However, this mechanism is difficult to predict because structures that share high sequence identity with AncLLysO were unavailable. In fact, the most similar structure to AncLLysO registered in PDB was AncLAAO and these structures share only 20% identity. Construction of an accurate homology model of AncLLysO is difficult, and therefore, we were required to determine crystal structures of AncLLysO to elucidate the mechanism.

**Figure 4:**
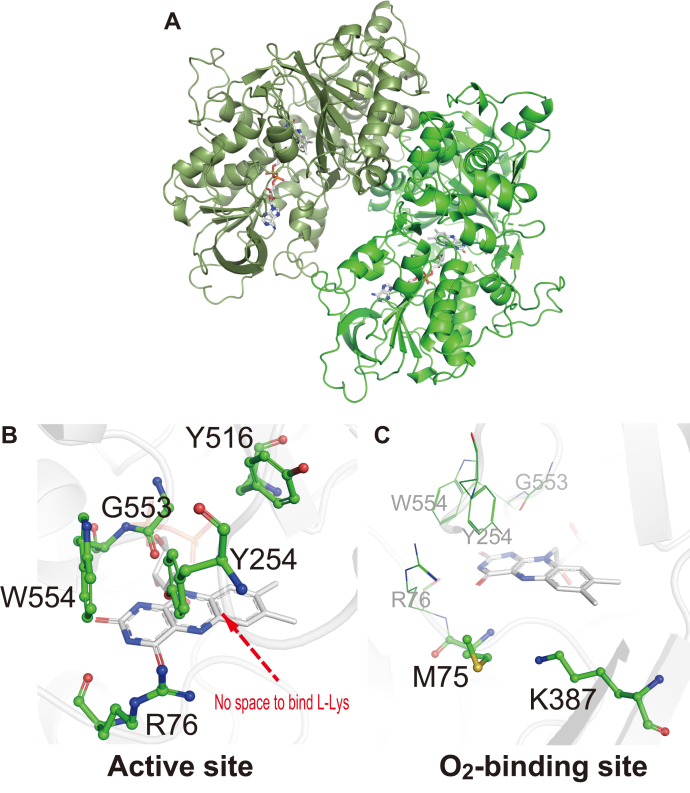
**Structure analysis of AncLLysO ligand-free form.** Overall structure of AncLLysO ligand-free form (*A*). Active site structure of AncLLysO(LF) (*B*). Side chain of Y254 occupies active site of AncLLysO, so there is no space to bind substrate, L-Lys. O_2_-binding site of AncLLysO (*C*). As well as the previously reported LAAO family, there is a Lys residue (K387) that coordinates O_2_ to oxidize FADH_2_.

In this study, the crystal structure for the ligand-free form of AncLLysO (AncLLysO(LF)) was determined at a 2.4-Å resolution. The initial phase was determined using the iodide single anomalous dispersion (SAD) method. Crystallographic parameters are represented in Table 2. AncLLysO has a fold typical of the flavin-dependent amine oxidase (FAO) superfamily (Fig. 4*A*)—the Rossmann-core fold, which recognizes FAD, and a hot-dog-like fold, which is important for the recognition of substrates (29). Structural analysis of AncLLysO(LF) by DALI server (46) also supported this point as the top ten structures that have structural similarity to AncLLysO are members of the FAO superfamily (Table 3).

**Table 2:** Statistics of X-ray diffraction data collection AncLLysO for native (ligand-free) and substrate-binding forms of K387A variant (L-Lys- and L-Arg-binding form)

Parameter	Native (ligand-free)	Iodide-SAD	L-Lys binding (K387A variant)	L-Arg binding (K387A variant)
Space group	P2_1_2_1_2_1_	P2_1_2_1_2_1_	P2_1_	P2_1_
Unit cell parameters				
a (Å)	89.4	90.3	85.8	85.9
b (Å)	80.5	80.6	80.1	80.1
c (Å)	173.8	172.8	88.0	88.0
α (degree)	90.0	90.0	90.0	90.0
β (degree)	90.0	90.0	90.4	90.5
γ (degree)	90.0	90.0	90.0	90.0
X-ray source	BL5A (PF)	BL5A (PF)	BL5A (PF)	NW12A (PF-AR)
Wavelength (Å)	1.00	1.70	1.00	1.00
Resolution (Å)	49.3–2.2 (2.31–2.20)	49.4–2.4 (2.53–2.40)	42.9–2.4 (2.53–2.40)	48.9–2.2 (2.32–2.20)
No. of reflections^a^	864,192	2,622,002	315,778	410,497
No. of unique reflections	63,996	50,279	46,762	60,710
Completeness (%)	98.8 (97.8)	100 (99.9)	99.8 (99.0)	99.7 (97.8)
I/sig(I)	18.9 (3.2)	42.3 (12.4)	16.2 (4.0)	15.2 (3.9)
CC_1/2_	0.999 (0.868)	1.00 (0.990)	0.998 (0.926)	0.998 (0.899)
*R*_merge_^b^	0.106 (0.846)	0.092 (0.405)	0.087 (0.421)	0.084 (0.446)
*B* of Wilson plot (Å)^2^	29.0	30.1	27.6	26.8
Iodide Sites		81		
FOM before DM^c^		0.28		
FOM after DM^c^		0.71		
*R*^d^	0.216		0.185	0.192
*R*_free_^e^	0.255		0.231	0.231
RMSD of geometry				
Bond length (Å)	0.007		0.007	0.007
Bond angle (degree)	1.498		1.460	1.438
Geometry				
Ramachandran outlier (%)	0.3		0.3	0.1
Ramachandran favored (%)	99.7		99.7	99.9
PDB code	7EIH		7EII	7EIJ

Abbreviation: SAD, single anomalous dispersion.

a Sigma cutoff was set to none (F > 0σF).

b *R*_merge_ = Σ_*h*_Σ_*i*_|*I*_*i*_(*h*)−<*I*(*h*)>|/Σ_*h*_*I*(*h*), where *I*_*i*_(*h*) is the *i*^th^ measurement of reflection *h*, and <*I*(*h*)> is the mean value of the symmetry-related reflection intensities. Values in brackets are for the shell of the highest resolution.

c FOM before/after DM means the figure of merit before/after density modification.

d *R* = Σ||*F*_*o*_| − |*F*_*c*_||/Σ|*F*_*o*_|, where *F*_*o*_ and *F*_*c*_ are the observed and calculated structure factors used in the refinement, respectively.

e *R*_free_ is the *R*-factor calculated using 5% of the reflections chosen at random and omitted from the refinement.

**Table 3:** Top ten structures that have structural similarity to AncLLysO (PDB ID: Ligand free) by Dali search against PDB25

Organism	Class	PDB code monomer	Z-score^a^	r.m.s.d.^b^	lali^c^	%id^d^
*Synthetic construct*	Ancestral L-amino acid oxidase	7C4M-A	32.1	2.5	506	25
*Chromobacterium violaceum*	Flavin-dependent L-tryptophan oxidase, VioA	6ESE-A	29.9	2.7	398	19
*Exiguobacterium sibiricum*	Protoporphyrinogen oxidase	3LOV-A	29.7	3.4	392	15
*Gloydius halys*	L-amino acid oxidase	1TDO-A	29.4	2.6	436	17
*Nicotiana tabacum*	Protoporphyrinogen oxidase	1SEZ-A	28.0	4.0	399	15
*Homo sapiens*	Renalase	3QJ4-A	27.8	2.8	312	16
*Pseudomonas syringae pv.tomato*	Amine oxidase	3KKJ-A	27.0	2.9	311	17
*Homo sapiens*	Lysine-specific histone demethylase 1	2X0L-A	26.1	2.9	399	17
*Homo sapiens*	Amine oxidase	2Z5X-A	25.9	3.2	422	14
*Homo sapiens*	Lysine-specific histone demethylase 1B	4FWE-A	25.6	3.1	399	17

a A measure of the statistical significance of the result relative to alignment of random structure.

b Root-mean-square deviation for Cα atoms.

c Number of aligned residues.

d Sequence identity between AncLLysO and the targeted chain.

The active site structure of AncLLysO(LF) indicated that residues that would form interactions with the main-chain L-Lys are present including R76, G553, and W554 (Fig. 4*B*). These residues are highly conserved in other LAAOs (asterisk in Fig. S2) (4). On the other hand, there is no space to bind L-Lys at the site as the side chain Y254 occupies the space (Fig. 4*B*), inferring that dynamic conformational changes of Y254 would be induced as the reaction progressed. The crystal structure of the L-Lys-binding form of AncLLysO has to be determined to prove this hypothesis, and inactivated variants are required for structure determination. We attempted to design the variants by mutating the residues at the O_2_-binding site (Fig. 4*C*). Previous research indicates that, in many FAD-dependent oxidases, the Lys residue at the site would recognize the O_2_ molecule, which oxidizes the FADH_2_, and their variants quietly reduce the activity (47). In AncLLysO, K387 corresponds with the residue (Fig. 4*C*), and the activity of the AncLLysO(K387A) variant was reduced below the detection limit of the activity assay as expected (Table 4). Utilizing AncLLysO(K387A), we attempted to determine the ligand-binding structures of AncLLysO.

**Table 4:** Enzyme kinetic parameters of AncLLysO variants toward L-Lys

Sample name	*k*_cat_	*K*_m_	*k*_cat_/*K*_m_
	s^−1^	mM	s^−1^ mM^−1^
AncLLysO	21.7 ± 0.9	0.3 ± 0.03	72.3
Residues that are located at O_2_-binding site			
K387A	N.D.	N.D.	N.D.
Residues that are located at substrate-recognition site			
R76A	N.D.	N.D.	N.D.
E383D	N.D.	N.D.	N.D.
Y268F	13.5 ± 1.4	1.0 ± 0.2	13.5
Y516F	9.3 ± 0.4	0.2 ± 0.03	46.5
Y516A	1.4 ± 0.05	4.1 ± 0.3	0.34
Residues that are located at plug loop			
G251A	10.4 ± 0.7	1.7 ± 0.3	6.1
G251P	1.1 ± 0.08	11.3 ± 1.3	0.09
Y253F	3.4 ± 0.2	0.15 ± 0.02	22.6
Y253A	0.21 ± 0.01	4.4 ± 0.3	0.05
Y254F	15.0 ± 1.1	0.6 ± 0.08	25
Y254A	6.0 ± 0.4	3.0 ± 0.5	2.0

The measurement of enzyme kinetic parameters was performed using two biological replicates per three technical replicates.

### Active site structures of the L-Lys- and L-Arg-binding forms of AncLLysO(K387A)

**Figure 5:**
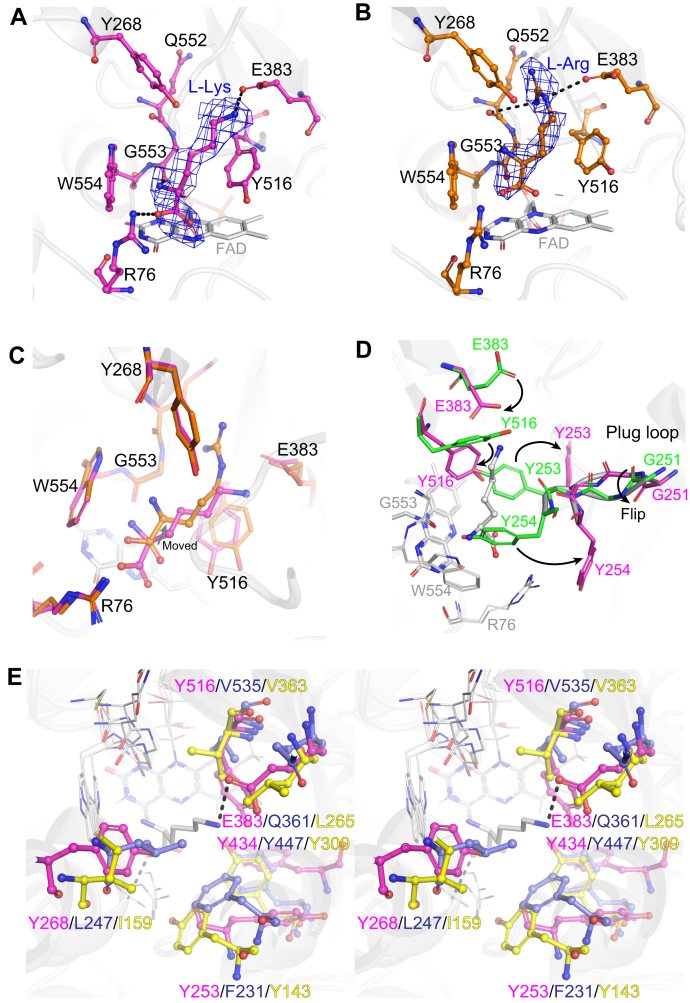
**Structure comparison at active site structures of ligand binding form of AncLLysO(K387A).** Active site structures for L-Lys- (*A*) and L-Arg- (*B*) binding form of AncLLysO(K387A). The L-Lys- and L-Arg-binding forms are *magenta* and *orange*, respectively. The polder Fo-Fc omit maps of L-Lys and L-Arg were contoured at 3.5 and 4.0 σ, respectively. Structure comparison of L-Lys- and L-Arg-binding forms of AncLLysO(K387A) (*C*). Conformational change of plug loop and active site residues by binding of L-Lys (*D*). AncLLysO(LF)- and L-Lys-binding forms of AncLLysO(K387A) are *green* and *magenta*, respectively. Structural comparison at the active site among AncLLysO, AncLAAO-N5 (PDB ID: 7C4N), and VioA (PDB ID: 6FW8) (*E*). The structures of the L-Lys-binding forms of AncLLysO(K387A), AncLAAO-N5, and VioA are represented by *magenta*, *dark blue*, and *yellow*, respectively.

Crystal structures of the L-Lys- and L-Arg-binding forms of AncLLysO(K387A) were determined at 2.4- and 2.2-Å resolution, respectively, using the cocrystallization method. In general, fitting of substrates into an electron density map would be difficult at the resolution of the AncLLysO(K387A) structure; however, the substrates appeared to be fitted into the map appropriately. In fact, the polder F_o_-F_c_ omit map indicates that L-Lys (Fig. 5*A*) and L-Arg (Fig. 5*B*) coordinate at the active site using different binding modalities; a stereo view of L-Lys- and L-Arg-binding forms is shown in Fig. S3. In the L-Lys-binding form, the carboxyl and side-chain amino groups form hydrogen bonds with R76 and E383, respectively (dotted line in Fig. 5*A*). In the L-Arg-binding form, only the guanidino group formed interactions with E383 and Q552 (dotted line in Fig. 5*B*). Differences in the binding mode would affect the substrate specificity of AncLLysO. In fact, the main-chain carbon atom of L-Arg is moved to the arrowed direction approximately 1.5 Å compared with L-Lys (Fig. 5*C*). Because of this movement, the distance between the main-chain carbon atom of L-Arg and the N5 atom of FAD is about 1.2 Å longer than in the case of L-Lys (Fig. 5*C*). This causes inefficient hydride transfer of Cα-H from L-Arg to the N5 atom of FAD during the reaction by AncLLysO. A similar phenomenon may occur in the L-Orn-binding form of AncLLysO.

Next, we attempted to represent structural changes of AncLLysO that are induced by the binding of substrates. Structural comparison between AncLLysO(LF)- (green) and the L-Lys-binding form of AncLLysO(K387A; magenta) are shown in Figure 5*D*, suggesting that dynamic structural changes could be confirmed at the following residues: 251 to 254, 383, and 516 (Fig. 5*D*). From a structural comparison between the ligand-free form and the L-Lys- or L-Arg-binding form of AncLLysO, substrate binding appeared to induce a conformational change of a loop that is formed by residues 251 to 254 (251-GGYY-254); *cis*–*trans* transformation at G251 is remarkable in this change. The loop position is consistent with a plug loop in L-LOX/MOG, which occurs when these two structures are superimposed on each other (40). Therefore, we call the loop a “plug loop” as well. The active site residues E383 and Y516 moved to the optimal position to recognize the L-Lys associating with the conformational change of the plug loop in AncLLysO (Fig. 5*D*).

Structural comparison between AncLLysO and other LAAOs may be helpful to predict how AncLLysO exhibits high substrate specificity toward L-Lys. Superimposed structures of AncLLysO(K387A), AncLAAO, and VioA are represented in Figure 5*D*, indicating that the size of the active site of AncLLysO is narrower than those of AncLAAO and VioA, as the site was formed mainly by bulky aromatic residues (Phe and Tyr). There are four aromatic residues in AncLLysO (magenta in Fig. 5*D*), whereas the number is decreased to two in AncLAAO and VioA (dark blue and yellow in Fig. 5*D*). The narrowing of the active site in AncLLysO made binding to amino acids other than L-Lys difficult; therefore, this could be one of the reasons that AncLLysO exhibits high specificity toward L-Lys.

### Enzyme kinetic analysis of AncLLysO variants

The structural analysis indicated that conformational rearrangement of the active site structure would be caused by the binding of substrates, triggering a dynamic conformational change of the plug loop. The next challenge is to demonstrate the functional role of the residues, which is related to the rearrangement using biochemical assays. To accomplish this, we attempted to analyze the enzyme kinetic parameters of several AncLLysO variants.

**Figure 6:**
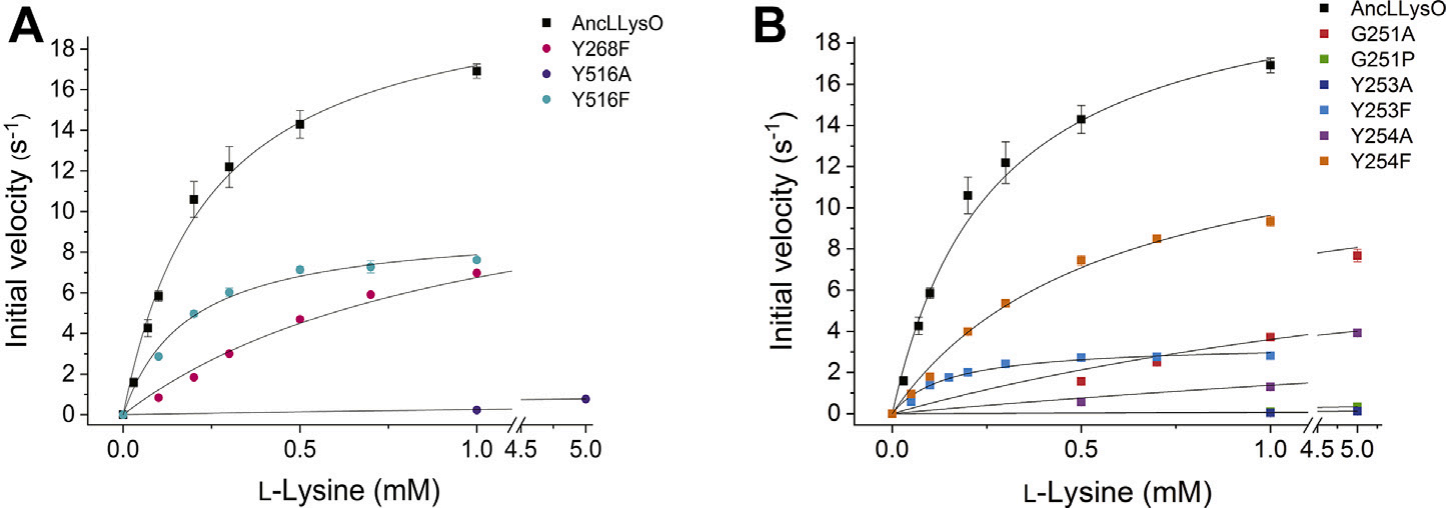
**Enzyme kinetics plots of AncLLysO variants.** The following two types of variants were designed to estimate the enzymatic functional roles of AncLLysO; one is the residue that is located near the substrate (*A*) and the other is the residue located on the plug loop (*B*). All of the experiments were performed using two biological replicates per three technical replicates.

First, analysis of AncLLysO variants of the substrate recognition sites (R76A, E383D, Y268F, and Y516F) was performed, and enzyme kinetic plots and parameters are represented in Figure 6*A* and Table 4, respectively. This analysis indicated that mutation of residues that form hydrogen bonds with L-Lys inactivated AncLLysO. In fact, the activity of R76A and E383D variants was too low to estimate their parameters (Table 4). On the other hand, activity loss by the mutations would be attenuated if hydrophobic interactions are maintained after the mutations; *k*_cat_/*K*_m_ values of Y268F and Y516F were approximately 20% and 55% that of AncLLysO (Table 4).

Next, an analysis of AncLLysO variants of the plug loop was performed. Kinetic plots and parameters are shown in Figure 6*B* and Table 4, respectively, suggesting that the side-chain phenyl groups of Y253 and Y254 are important for AncLLysO to express high activity. In fact, *k*_cat_ and *k*_cat_/*K*_m_ values were largely decreased by mutating the residues to Ala compared with Phe (Table 4). In the variants, the decrease of the Y253A variant was remarkable; the *k*_cat_ and *k*_cat_/*K*_m_ values were two and four orders lower than those of AncLLysO (Table 4). For the G251 variants, loss of activity could be confirmed as the residue was located separately from the active site. The decrease of the G251P variant was particularly apparent; the *k*_cat_ and *k*_cat_/*K*_m_ values were approximately 19- and 800-fold lower than those of AncLLysO (Table 4). Here, mutation of G251 to Pro increases the energy barrier required to switch between the *cis* and *trans* forms; in fact, the energy required to induce *cis*–*trans* isomerization of Pro was estimated to be approximately 20 kJ/mol (48). This suggested that G251P would fix its conformation only in the *cis* or *trans* form. In this situation, the conformational change at the plug loop observed in Figure 5*D* was suppressed, and recognition of L-Lys and/or emission of the product may be inhibited in the G251P variant.

Enzyme kinetics of the monooxygenase activity of AncLLysO can be estimated by quantifying the amount of 5-APNM produced by the aminoamide oxidizing enzyme from *Aspergillus carbonarius* AIU 205 (AcAOx) (49). Referring to the previous study (40), enzyme kinetics for the monooxygenase activity of AncLLysO were assayed utilizing the purified AcAOx as the quantifying enzymes of 5-APNM. However, the concentration of 5-APNM could not be quantified by the assay because the amount of 5-APNM produced by AncLLysO was too low to detect with AcAOx. In fact, for L-LOX/MOG, of which enzyme kinetic parameters could be estimated by the assay, the main product of the reaction with L-Lys was 5-APNM (85% for 5-APNM *versus* 0.32% for 5-APNA), whereas the main product for AncLLysO was 5-APNA (3.2% for 5-APNM *versus* 66% for 5-APNA, Fig. 3).

## Discussion

**Figure 7:**
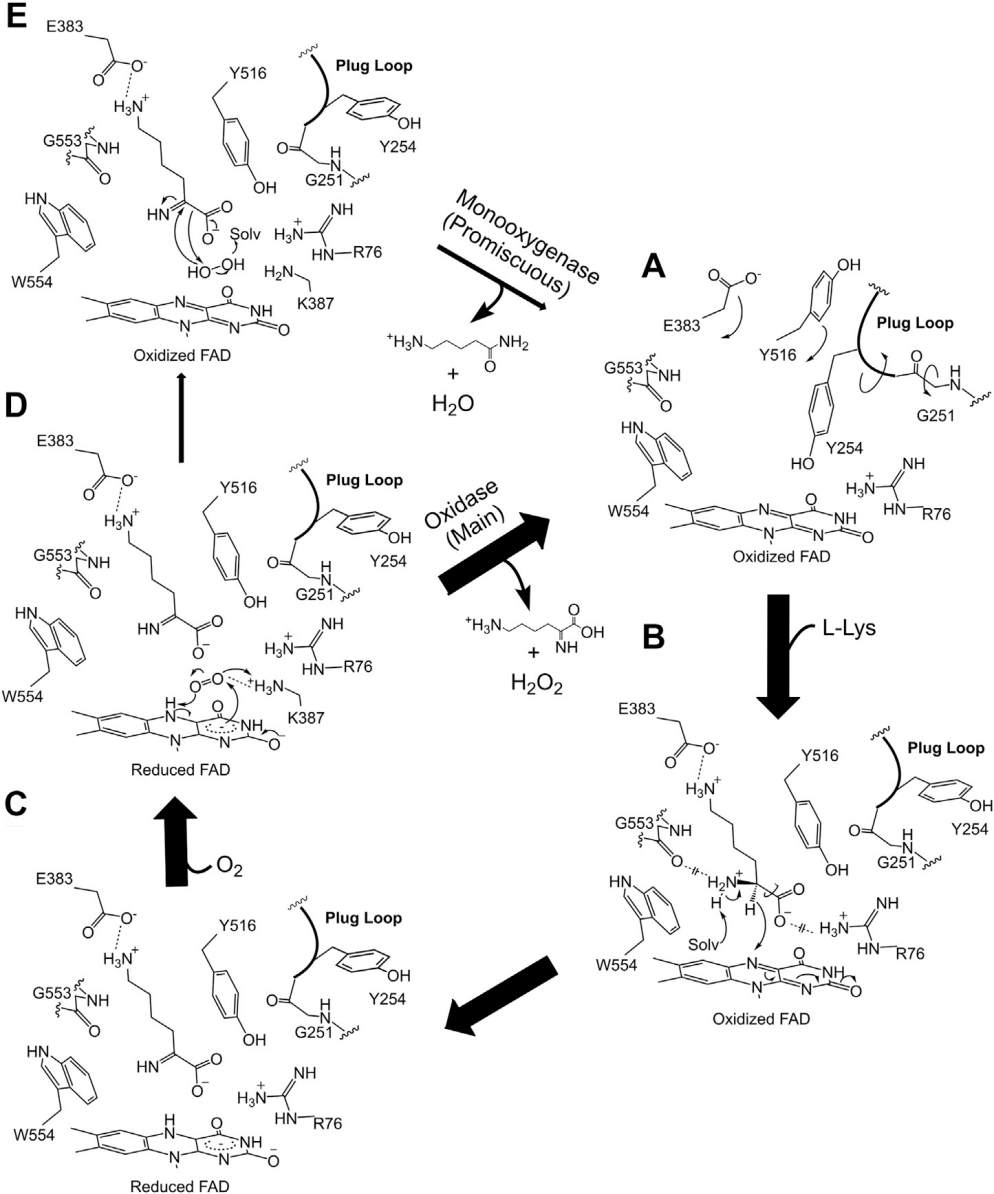
**Proposed reaction mechanism of AncLLysO for L-Lys α-oxidase and monooxygenase activity.** In this scheme, ligand free, ligand binding, and product binding form were represented as *A*, *B*, and *C*, respectively. After oxidation of an FADH (*D*), the product imino acid and H_2_O_2_ were released into the solvent (*D* to *A*); this is main oxidase activity. On the other hand, 5-APNM and H_2_O were released into the solvent via state *E*; this is promiscuous monooxygenase activity confirmed in AncLLysO.

Summarizing the results for biochemical and structural analysis of AncLLysO and their variants, we propose the reaction mechanism shown in Figure 7. In the ligand-free form, a cavity at the active site to recognize substrates is occupied by the side-chain Y254, which is the residue forming the plug loop (A in Fig. 7); this occupation would bring about structural changes of the substrate recognition residues, such as E383 and Y516 and made it difficult to predict the recognition mechanism solely from the structure of the AncLLysO ligand-free form. Rearrangement of the active site structure would be induced by the binding of L-Lys. Specifically, the side-chain Y254 moved away from the active site, associating with the *cis*–*trans* transformation of G251, and simultaneously the side chains E383 and Y516 flipped into the active site to recognize L-Lys (A to B in Fig. 7). The *k*_cat_/*K*_m_ value of Y516A decreased as the *K*_m_ value increased. This did not occur with the Y516F variant (Table 4), suggesting that the side-chain phenyl group of Y516 is important to form the substrate-binding site that recognizes L-Lys more efficiently. The functional importance of the structural change of the plug loop was shown by enzyme kinetics analysis (Table 4). All of the variants on the plug loop decreased the *k*_cat_/*K*_m_ value compared with AncLLysO despite no residues forming interactions with L-Lys in the ligand-binding form (Table 4 and Fig. 5).

AncLLysO oxidizes L-Lys *via* an identical mechanism already described for LAAOs (4). The main-chain amino group of L-Lys is deprotonated by an activated solvent water molecule, and the hydride on the main-chain carbon atom is transferred to FAD (B to C in Fig. 7). The oxygen molecule would bind to the active site by forming an interaction with the side-chain amino group of K387, and the oxidation of reduced FAD progressed continuously (D in Fig. 7). To predict the oxidation mechanism, we referred to the study of D-amino acid oxidase, which catalyzes oxidation of D-amino acids through a mechanism similar to AncLLysO (50). From the LC-HRMS analysis, the produced imino acid is converted to 5-APNA or 5-APNM *via* a different mechanism. For 5-APNA production, the imino acid and H_2_O_2_ are released to the solvent without reacting to each other (D to A in Fig. 7). The released product is hydrolyzed and decarboxylated to 5-APNA. The mechanism generally corresponds to that of LAAOs (4). On the other hand, 5-APNM is produced by the monooxygenase activity of AncLLysO *via* a mechanism similar to that of L-LOX/MOG (41). Utilizing H_2_O_2_, imino acid is decarboxylated to 5-APNM at the active site (D to E in Fig. 7). Trisrivirat *et al.*, (41) reported that a plug loop is required to exhibit the activity in L-LOX/MOG, and AncLLysO also bears this plug loop (251-GGYY-254). Although enzyme kinetic parameters for monooxygenase activity of AncLLysO and their variants could not be determined because of their promiscuous nature, the mutation at the loop may affect the monooxygenase activity. Here, AncLLysO shared low sequence identity with L-LOX/MOG (<20%), but they exhibited similar activity. In other LAAOs, the plug loop also exists (filled circle in Fig. S2), suggesting that monooxygenase activity may be confirmed in other LAAOs that belong to an evolutionally distant family, similar to the case of AncLLysO and L-LOX/MOG.

Currently, four enzymes that belong to the FAO superfamily and exhibit LAAO activity are reported. These include AncLAAO (26, 28), VioA (31), AROD (27, 30), and AncLLysO. These enzymes share low sequence identity to each other and to other proteins in the superfamily registered in the PDB database (<30%). Their substrate selectivity and specificity were clearly different from each other. However, their overall structures were highly conserved and the enzymes could oxidize common substrates. For example, L-Lys and L-Arg were oxidized by AncLAAO, AROD, and AncLLysO, and L-Trp was oxidized by VioA and AncLAAO. Based on previous research of enzyme evolution (51), we predicted that the four enzymes would be derived from a common ancestor, which may exhibit broad substrate selectivity toward L-amino acids, like AncLAAO. The promiscuous enzyme functions of the current enzymes would be a functional vestige of the progenitor enzymes; therefore, it is reasonable to assume that the common ancestors of the four enzymes bear monooxygenase activity similar to AncLLysO. These enzymes could be investigated in the near future.

## Conclusion

Taken together, we assigned a novel FAD-dependent enzyme, called LLysO and bearing a main L-Lys α-oxidase and promiscuous monooxygenase activity, from the database. Here, LLysO has a substrate specificity more specifically toward L-Lys than the L-LOX/MOG. The *k*_cat_/*K*_m_ value of L-LOX/MOG was equivalent to L-Lys and L-Orn (40), whereas, for AncLLysO and CaLLysO, the *k*_cat_/*K*_m_ values toward L-Lys were greater than 15-fold higher than the values toward L-Orn (Table 1). The assignment of LLysO was achieved by the combinational usage of the sequence classification method utilizing correlatively mutated residues as the motif and ancestral sequence reconstruction. Although sequence identity among AncLLysO, L-LOX/MOG, and TvLLysO was quite low (<30%), the interaction mode is highly conserved with L-Lys. The acidic amino acids (Asp or Glu) and Arg at the active site formed a hydrogen bond with the side-chain amino group and the main-chain carboxyl group of L-Lys, respectively (Fig. S4, *A*–*C*). This suggests that, as in the case of the assignment of AncLLysO, there is a chance to find new LAAOs that have unique sequences compared with previously reported LAAOs.

AncLLysO has several characteristics that enable its enzymatic functions to be revealed by experimental approaches. These include high thermostability, resilience against mutations, and ease of crystallization, all of which allowed us to reveal the catalytic mechanism of AncLLysO at the molecular level. In the future, new enzymes could be screened from databases in a similar manner and their functions characterized by applying the combinational approach reported in this study.

## Experimental procedures

### Reconstruction of AncLLysO

A total of 28 sequences (Table S1) that were selected by applying the procedure described in Figure 1 and one sequence bearing low identity (approximately 20%) compared with the selected sequences were aligned by MAFFT software (52). The aligned sequences were analyzed by MEGA6 (53), and phylogenetic tree data were prepared using the maximum-likelihood method. The aligned sequences and the tree data were submitted to the FastML web server (54) and JTT empirical models were adopted for the analysis. Finally, we selected a common ancestral sequence generated by the FastML as AncLLysO.

### Overexpression and purification of AncLLysO and CaLLysO

Plasmids containing AncLLysO and CaLLysO were digested with *Nco*I and *Xho*I, and corresponding DNA sequences were subcloned into the pET28a vector, which were cut with the same two restriction enzymes. DNA encoding LLysOs was synthesized by GENEWIZ. The produced expression plasmids were transformed into the *E. coli* strain BL21(DE3). The strain was cultivated in 1 l of LB broth containing 30 μg/ml of kanamycin at 37 °C. The temperature was lowered to 18 °C when the *A*_600_ value reached 0.6 to 0.8, and then isopropyl-β-D-thiogalactopyranoside was added to a final concentration of 0.5 mM. After the strains were cultivated overnight, they were collected by centrifugation. The collected cells were suspended into bufferA (20 mM Tris-HCl [pH 8.0] and 10 mM NaCl). After sonication of the cells, the supernatant was collected with centrifugation at 11,000*g* for 40 min. The supernatant was applied to a HisTrap-HP column (GE Healthcare), and the column was washed with 30 ml of bufferA containing 10 mM imidazole. The samples were eluted by 15 ml of bufferA containing 30 mM imidazole. The samples were applied to a MonoQ column (GE Healthcare) that was equilibrated by bufferA and purified by linear gradient utilizing bufferA and bufferB (20 mM Tris-HCl [pH 8.0] and 500 mM NaCl) as elution buffer. The fractions exhibiting the highest *A*_450_/*A*_280_ ratio were collected and concentrated to 500 μl. The concentrated samples were applied to a Superdex 200pg column that was equilibrated with bufferA; the purity was confirmed by SDS-PAGE. The purified samples were utilized in subsequent analysis.

UV-visible spectra of AncLLysO and the variants were measured to estimate FAD contents using the *A*_280_/*A*_450_ ratio for each sample. The analysis indicated that, for all of the AncLLysO variants generated in this study, the contents reached greater than 80%, indicating that differences in contents had a minimal effect on the relative comparison of enzyme kinetics parameters (Table 4).

### Site-directed mutagenesis of AncLLysO

Plasmids containing AncLLysO cloned into pET28b were utilized as a template. Site-directed mutagenesis was performed utilizing QuikChange Lightning Multi-site mutagenesis kit (Agilent Technologies). Primers utilized to design the variants are listed in Table S3. Sequence confirmation of the AncLLysO variants was performed by DNA sequencing.

### Analysis of substrate selectivity, thermal stability, and enzyme kinetics of AncLAAO and CaLLysO

Oxidase activity of CaLLysO, AncLLysO, and their variants was measured by quantifying the concentration of H_2_O_2_ produced by the enzymatic reaction utilizing a color-developed method. The components of the assay buffer were as follows: 10 mM amino acid, 1.5 mM 4-aminoantipyrine, 2 mM phenol, 50 U/ml horseradish peroxidase, and 100 mM buffer. The following four types of buffers and 10 mM L-Lys were utilized in the assay to estimate optimal pH value: sodium acetate (pH 3.5–4.5), Bis-Tris-HCl (pH 6.0–7.0), Tris-HCl (pH 7.0–8.5), and BICIN (pH 9.0). Substrate selectivity was estimated utilizing the following amino acid and buffer: 10 mM amino acids and 100 mM Bis-Tris-HCl (pH 7.0). Thermostability was measured utilizing the assay buffer containing 10 mM L-Lys and 100 mM Bis-Tris-HCl (pH 7.0). The initial velocity of AncLLysO and CaLLysO was calculated by monitoring the time-dependent absorption change at 505 nm, which was derived from the produced pigment bearing ε_505_ = 12,700 M^−1^ cm^−1^ with UV-visible spectrometer (UV-2450, Shimadzu).

The kinetic parameters of CaLLysO, AncLLysO, and their variants toward L-Lys, L-Arg, and L-ornithine (L-Orn) were measured under the conditions containing the following concentrations of substrates: 0.1 to 1.0 mM L-Lys, 1.0 to 10 mM L-Arg, and 5.0 to 50 mM L-Orn, respectively. A procedure identical to the measurement of substrate selectivity was applied to determine initial velocity. The enzyme kinetic parameters were estimated by fitting the initial velocity to the Michaelis–Menten equation with the nonlinear least-squares method by ORIGIN software; the parameters are represented in Table 1. All of the experiments were performed using two biological replicates per three technical replicates.

### Analysis of products by LC-HRMS

LC-HRMS analysis was performed using Q Exactive (Thermo Fisher Scientific), equipped with an electrospray ionization module. Here, the following columns, which are joined to the system, were utilized to detect the products by LC-HRMS: a UPLC column (XBridge BEH Amide XP column [length, 2.1 × 50 mm^2^; inner diameter (i.d.), 2.5 μm; Nihon Waters K.K.]) equipped with a guard column (XBridge BEH Amide XP VanGuard cartridge [length, 2.1 × 5 mm^2^; i.d., 2.5 μm; Nihon Waters K.K.]). The column was kept at 40 °C. The volume of injected samples was 1 μl. The following two solutions were utilized as the mobile phase: 5 mM ammonium formate and 90% (v/v) acetonitrile (solution A) and 5 mM ammonium formate and 50% (v/v) acetonitrile (solution B). The flow rate was set to 0.4 ml/min, and the products were eluted by applying the following conditions: (1) 0% B for 1 min, (2) 0% to 100% B over 4 min, (3) 100% B for 2 min, and (4) 0% B for 5 min.

### Crystallization and X-ray data collection of AncLLysO

The purified AncLLysO was concentrated to about 15 mg/ml by centrifugation. Crystallization of AncLLysO for phase determination was performed as follows. After mixing a total of 1.5 μl of the concentrated AncLLysO samples with 1.0 μl of reservoir solution, composed of 12.5% (w/v) PEG3350, 0.1 M Hepes-NaOH (pH 7.5), and 0.2 M ammonium sulfate, the AncLLysO crystals appeared at 22 °C. The crystals were soaked in a cryoprotectant reservoir quickly (25% [w/v] PEG3350, 0.1 M Hepes-NaOH [pH 7.5], 0.2 M ammonium sulfate, 20%[v/v] glycerol, and 0.2 M NaI), and the crystals were flash-cooled under a liquid nitrogen stream (100 K). X-ray diffraction data were collected using a Pilatus3 detector instrument at a BL5A beamline in the Photon Factory. Integration and scaling of the data were performed by XDS (55) and SCALA (56), respectively. The initial phase determination was achieved by the iodide single anomalous dispersion method. AutoSol, implemented in PHENIX software (57), assigned a total of 81 anomalous sites by analyzing the data. Model building was performed by AutoBuild (57) and Coot (58), and the initial structure for the ligand-free form of AncLLysO could be obtained.

Crystallization of the L-Lys- and L-Arg-binding forms of AncLLysO was performed by following procedures utilizing the AncLLysO(K387A) variant as a sample. The samples were concentrated to approximately 15 mg/ml. Samples that bind L-Lys or L-Arg to the active site were prepared by mixing a total of 90 μl of the concentrated samples with 10 μl of 100 mM L-Lys and 100 mM L-Arg, respectively. Crystals could be obtained by mixing a total of 1.5 μl of the samples with 1.0 μl of reservoir solution (25% [w/v] PEG3350 and 0.1 M Tris-HCl [pH 8.5]) and incubating at 22 °C. The crystals were soaked in a cryoreservoir (25% [w/v] PEG3350, 0.1 M Tris-HCl [pH 8.6], 10 mM NaCl, and 20% [v/v] ethylene glycol) containing 10 mM L-Lys or L-Arg for 60 min. The soaked crystals were flash-cooled under a cryonitrogen stream (100 K). X-ray diffraction data were collected at BL5A in the Photon Factory. Data integration and scaling were performed by XDS and SCALA, respectively, and the phase was determined by the MOLREP software (59) utilizing the structure of the ligand-free form of AncLLysO as a template. Model building and refinement were performed by COOT (58) and either REFMAC (60) or PHENIX (57), respectively. All figures were prepared by PyMOL (61). Crystallographic parameters are represented in Table 2.

## Data availability

All data are contained within this article.

## Supporting information

This article contains supporting information.

## Conflict of interest

The authors declare that they have no conflicts of interest related to the contents of this article.
