# Supplementary material for: Catalytic mechanism of ancestral L-lysine oxidase assigned by sequence data mining
Source: J Biol Chem. 2021 Aug 4;297(3):101043. doi: 10.1016/j.jbc.2021.101043 (PMC8405998; doi:10.1016/j.jbc.2021.101043)
Supplement: Supplemental Figures S1–S4 and Tables S1–S3 [file mmc1.pdf]

Catalytic Mechanism of Ancestral L-Lysine Oxidase Assigned by Sequence Data Mining

Sayaka Sugiura[1, 4], Shogo Nakano[1, 3, 4, *], Masazumi Niwa[1], Fumihito Hasebe[1], Daisuke Matsui[2] and Sohei Ito[1]

[1]Graduate Division of Nutritional and Environmental Sciences, University of Shizuoka, 52-1 Yada, Suruga-ku, Shizuoka 422-8526, Japan

[2]Department of Biotechnology, College of Life Sciences, Ritsumeikan University, 1-1-1 Noji-higashi, Kusatsu, Shiga 525-8577, Japan

[3] PREST, Japan Science and Technology Agency, Kawaguchi, 332-0012, Japan

[*]To whom correspondence may be addressed: Graduate Division of Nutritional and Environmental Sciences, University of Shizuoka, 52-1 Yada, Suruga-ku, Shizuoka 422-8526, Japan, Tel.: +81-54-264-5538 (Ext.), E-mail: snakano@u-shizuoka-ken.ac.jp

[4]These authors contributed equally to this work.

Keywords: L-amino acid oxidase, crystal structure, ancestral sequence reconstruction, deracemization

Contents

・**Supporting Figures** **S3-S6**

Distribution of conservation energies and sequence logo S3

Multiple sequence alignment of five different LAAOs S4

Stereo view of L-Lys (A) and L-Arg (B) binding forms of AncLLysO(K387A) variant S5

Active site structures of three LAAOs which can oxidize L-Lys S6

・**Table contents** **S7-S9**

Protein sequences which are utilized to design AncLLysO S7

Protein sequence of AncLLysO and CaLLysO S8

Primers to design AncLLysO variants S9

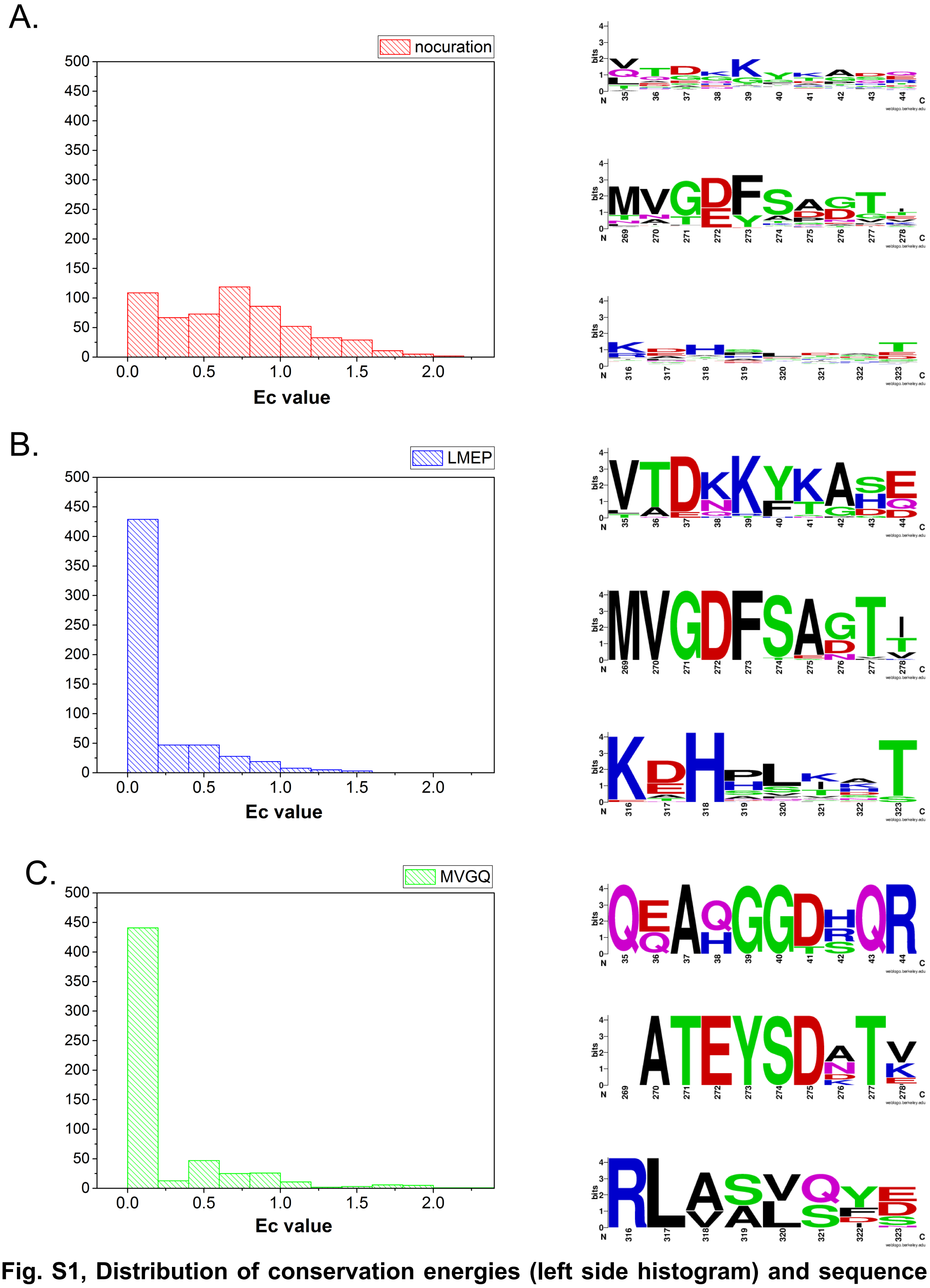

Fig. S1, Distribution of conservation energies (left side histogram) and sequence logo (right side) of no-curated (total A), LMEP (B) and MVGQ (C) libraries, respectively.

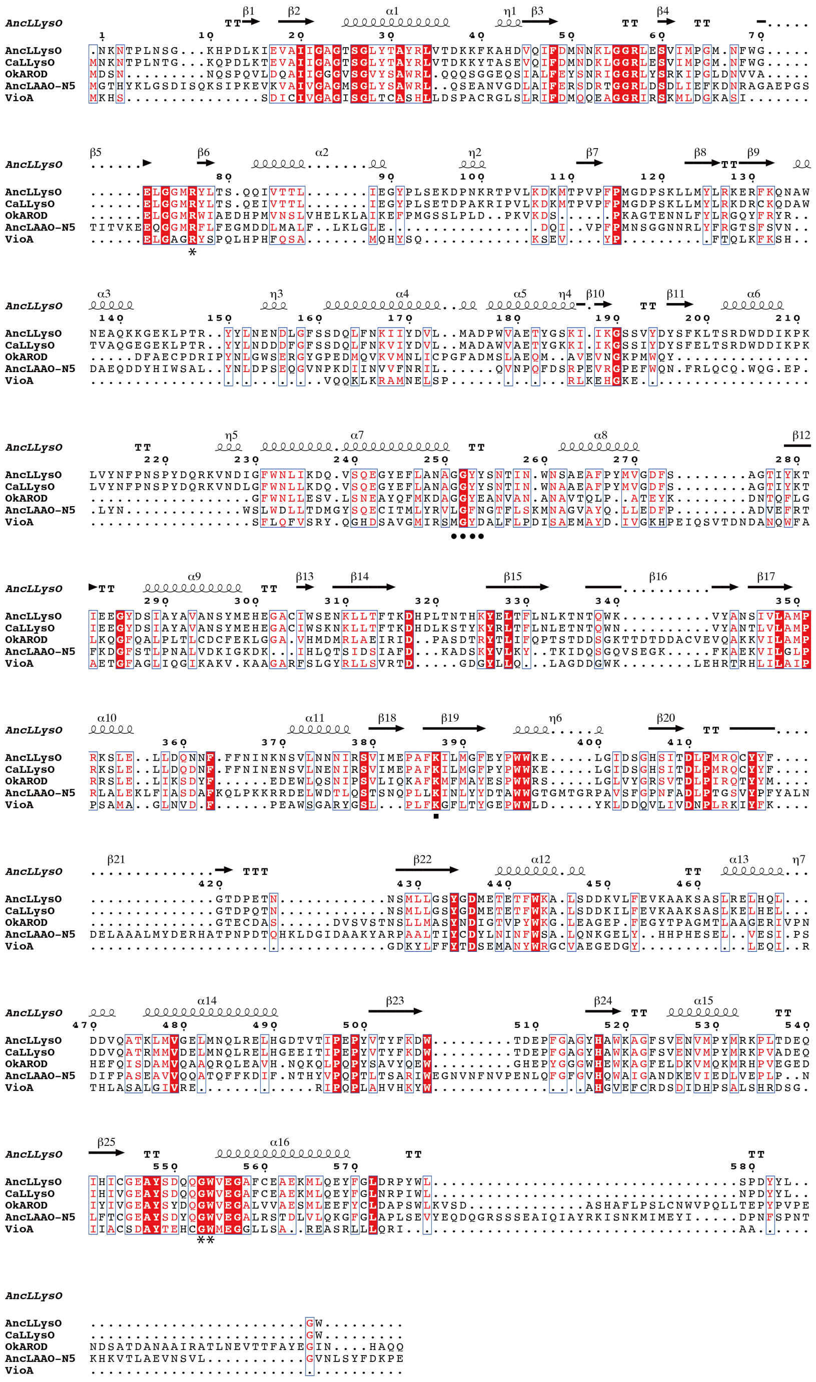

Fig. S2. Multiple sequence alignment of five different LAAOs. Asterisk (*) represents the residues recognizing main chain amino acids, filled square (■) represents O_2 recognition residue, and filled circle (•) represents the residues forming plug loop, respectively. The sequences for AncLLysO and CaLLysO were indicated in Table S2. Sequences of AncLAAO-N5, OkAROD and VioA were registered in PubMed as 7C4N_A, WP_028295159.1, and WP_011136821.1, respectively.

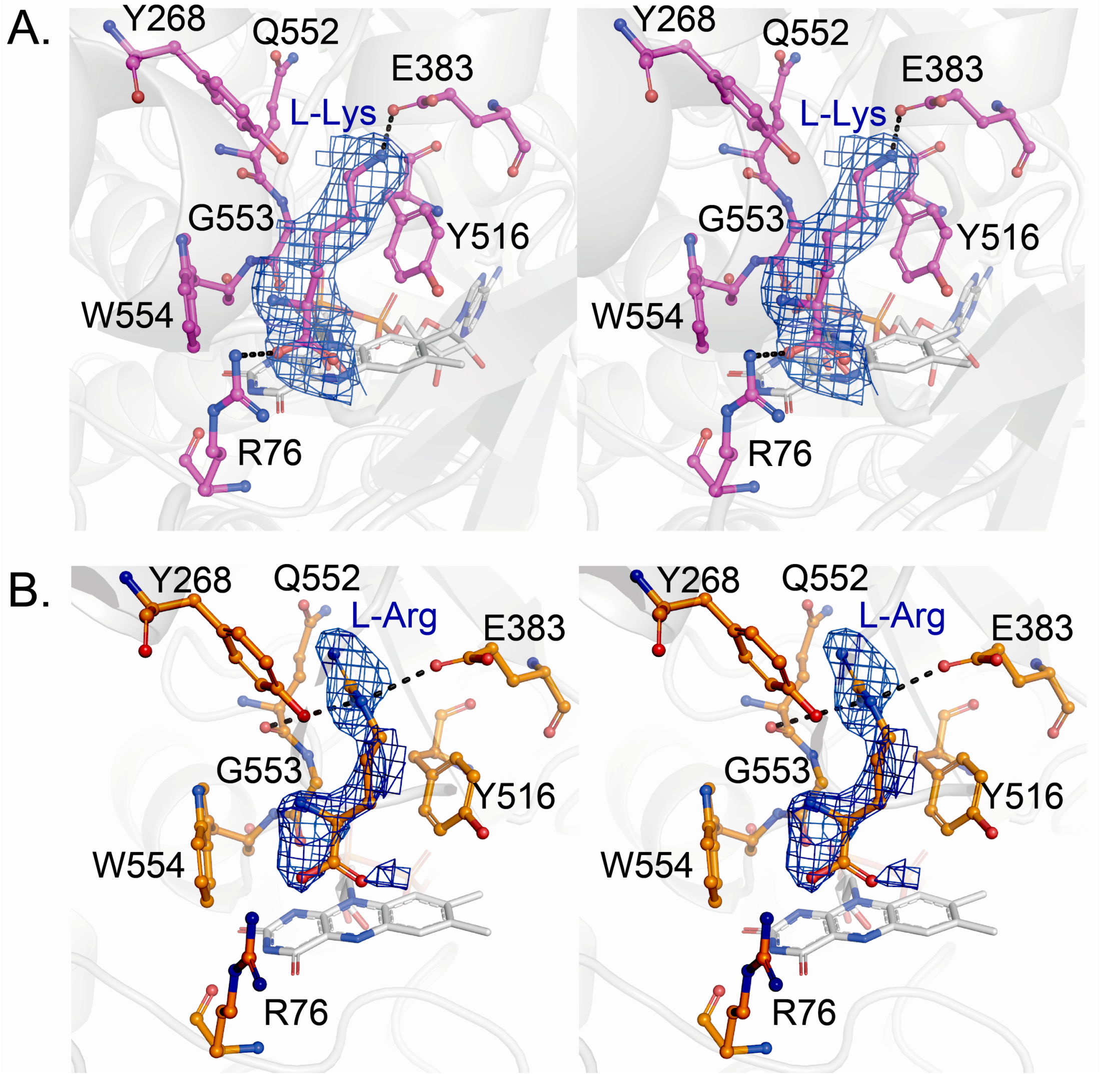

Fig. S3. Stereo view of L-Lys (A) and L-Arg (B) binding forms of AncLLysO(K387A) variant. The polder F_o-F_c omit maps of L-Lys and L-Arg were contoured at 3.5 and 4.0σ, respectively.

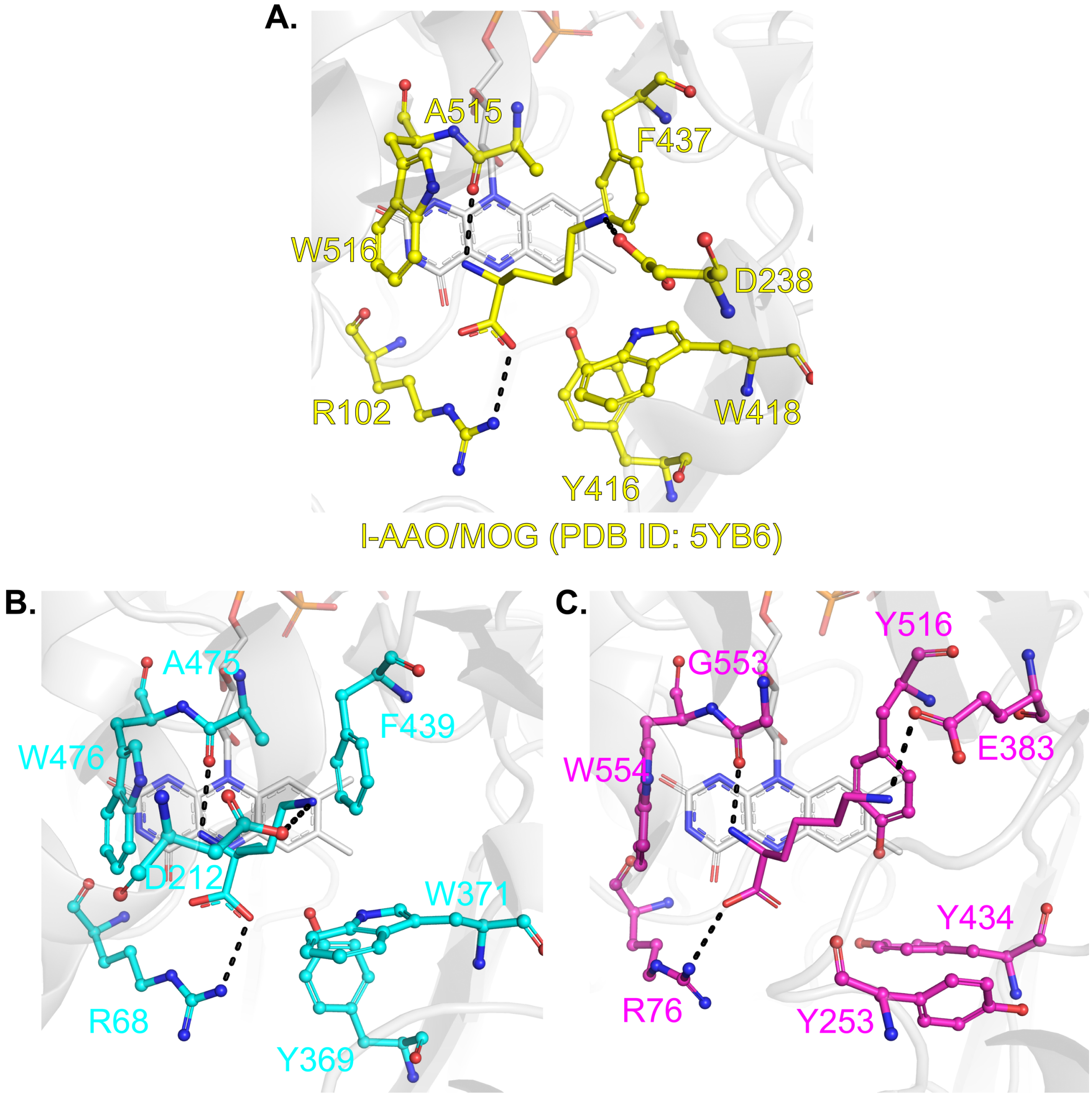

l-AAO/MOG (PDB ID: 5YB6)

TvLysOX (PDB ID: 7C3H)

AncLLysO

Fig. S4. Active site structures of three LAAOs which can oxidize L-Lys. Here, the structures for l-AAO/MOG (A), TvLysOX (B) and AncLLysO (C) were represented as yellow, cyan and magenta, respectively. The hydrogen bonds formed between active site residues and L-Lys were shown as dotted lines.

Table S1. 28 homolog sequences to AncLAAO-N5 that were utilized to design ancestral L-lysine alpha-oxidase (AncLLysO)

Accession	Locus tag	Species
WP_055979056.1	FAD-dependent oxidoreductase	*Chryseobacterium* sp. Leaf405
WP_025142427.1	FAD-dependent oxidoreductase	*Pedobacter jeongneungensis*
WP_124559759.1	FAD-dependent oxidoreductase	*Pedobacter* sp. KBW01
WP_062648590.1	FAD-dependent oxidoreductase	*Chryseobacterium kwangjuense*
OJV73420.1	monoamine oxidase	*Flavobacterium* sp. 40-81
WP_026706586.1	FAD-dependent oxidoreductase	*Flavobacterium soli*
WP_120230750.1	FAD-dependent oxidoreductase	*Chryseobacterium* sp. AG363
WP_068845637.1	FAD-dependent oxidoreductase	*Flavobacterium chilense*
WP_105683457.1	FAD-dependent oxidoreductase	*Chryseobacterium culicis*
WP_073412263.1	FAD-dependent oxidoreductase	*Flavobacterium defluvii*
WP_110835007.1	FAD-dependent oxidoreductase	unclassified *Flavobacterium*
WP_115848208.1	FAD-dependent oxidoreductase	unclassified *Flavobacterium*
WP_047423267.1	FAD-dependent oxidoreductase	*Chryseobacterium* sp. YR480
WP_077414784.1	FAD-dependent oxidoreductase	*Chryseobacterium* sp. JV274
WP_124537106.1	FAD-dependent oxidoreductase	*Chryseobacterium* sp. KBW03
WP_136402114.1	FAD-dependent oxidoreductase	*Flavobacterium* sp. CC-CTC003
WP_135526289.1	FAD-dependent oxidoreductase	*Flavobacterium* sp. DS2-A
WP_047440975.1	FAD-dependent oxidoreductase	*Chryseobacterium* sp. YR561
WP_047401021.1	FAD-dependent oxidoreductase	*Chryseobacterium* sp. YR460
WP_031457051.1	FAD-dependent oxidoreductase	*Flavobacterium chungangense*
WP_105704158.1	FAD-dependent oxidoreductase	*Chryseobacterium* sp. MYb7
WP_089481723.1	FAD-dependent oxidoreductase	*Flavobacterium araucananum*
WP_048506397.1	FAD-dependent oxidoreductase	*Chryseobacterium angstadtii*
WP_142722188.1	FAD-dependent oxidoreductase	*Chryseobacterium* sp. ON_d1
HCN50300.1	monoamine oxidase	*Chryseobacterium* sp.
WP_129536913.1	FAD-dependent oxidoreductase	*Chryseobacterium* sp. 3008163
WP_126650316.1	FAD-dependent oxidoreductase	*Chryseobacterium* sp. 17S1E7
WP_089756798.1	FAD-dependent oxidoreductase	*Chryseobacterium soldanellicola*

Table S2. Protein sequences of AncLLysO and CaLLysO

>CaLLysO

MNKNTPLNTGKQPDLKTEVAIIGAGTSGLYTAYRLVTDKKYTASEVQIFDMNDKLGGRLE
SVIMPGMNFWGELGGMRYLTSQEIVTTLIEGYPLSETDPAKRIPVLKDKMTPVPFPMGD
PSKLLMYLRKDRCKQDAWTVAQGEGEKLPTRYYLNDDDFGFSSDQLFNKVIYDVLMAD
AWVAETYGKKIIKGSSIYDYSFELTSRDWDDIKPKLVYNFPNSPYDQRKVNDLGFWNLLK
DQVSQEGYEFLANAGGYYSNTINWNAAEAFPYMVGDFSAGTIYKTIEEGYDSIAYAVAN
SYMEHEGACIWSKNKLLTFTKDHDLKSTYKYRLTFLNLETNTQWNVYANTLVLAMPRKS
LELLDQDNFFFNINENSVLNENIRSVIMEPAFKILMGFPYPWWKELGIDSGHSITDLPMR
QCYYFGTDPQTNNSMLLGSYGDMETETFWKALSDDKILFEVKAAKSASLKELHELDDV
QATRMMVDELMNQLRELHGEEITIPEPYVTYFKDWTDEPFGAGYHAWKAGFSVENVM
PYMRKPVADEQIHIVGEAYSDQQGWVEGAFCEAEKMLQEYFGLNRPIWLNPDYYLGW

>AncLLysO

MNKNTPLNSGKHPDLKIEVAIIGAGTSGLYTAYRLVTDKKFKAHDVQIFDMNNKLGGRLE
SVIMPGMNFWGELGGMRYLTSQQIVTTLIEGYPLSEKDPNKRTPVLKDKMTPVPFPMG
DPSKLLMYLRKERFKQNAWNEAQKKGEKLPTRYYLNENDLGFSSDQLFNKIIYDVLMAD
PWVAETYGSKIIKGSSVYDYSFKLTSRDWDDIKPKLVYNFPNSPYDQRKVNDIGFWNLIK
DQVSQEGYEFLANAGGYYSNTINWNSAEAFPYMVGDFSAGTIYKTIEEGYDSIAYAVAN
SYMEHEGACIWSENKLLTFTKDHPLTNTHKYELTFLNLKTNTQWKVYANSIVLAMPRKSL
ELLDQNNFFFNINKNSVLNNNIRSVIMEPAFKILMGFEYPWWKELGIDSGHSITDLPMRQ
CYYFGTDPETNNSMLLGSYGDMETETFWKALSDDKVLFEVKAAKSASLRELHQLDDVQ
ATKLMVGELMNQLRELHGDTVTIPEPYVTYFKDWTDEPFGAGYHAWKAGFSVENVMP
YMRKPLTDEQIHICGEAYSDQQGWVEGAFCEAEKMLQEYFGLDRPYWLSPDYYLGW

*These two sequences share about 90% sequence identity each other.

Table S3. Primer list to prepare site-directed variants of AncLLysO

Primers	
M75A	5’- AACTGGGTGGC**GCG**CGTTACCTGACCAGC -3’
R76A	5’- GGTGGCATG**GCT**TACCTGACCAGCCAGCAA -3’
G251A	5’- TTTCTGGCGAACGCG**GCT**GGCTACTATAGC -3’
G251P	5’- GAATTTCTGGCGAACGCG**CCT**GGCTACTAT -3’
Y253F	5’- GCGGGTGGC**TTC**TATAGCAACACCATCAAC -3’
Y253A	5’- GCGGGTGGC**GCC**TATAGCAACACCATCAAC -3’
Y254F	5’- GGTGGCTAC**TTT**AGCAACACCATCAACTGG -3’
Y254A	5’- GGTGGCTAC**GCT**AGCAACACCATCAACTGG -3’
Y268F	5’- GCGTTTCCG**TTC**ATGGTTGGTGACTTCAGC -3’
E383D	5’- GTTATTATG**GAT**CCGGCGTTTAAGATCCTG -3’
K387A	5’- AACCG**GCG**TTTGCGATCCTGATGGGTTTC -3’
D436E	5’- AGCTATGGC**GAG**ATGGAGACCGAAACCTTT -3’
Y516F	5’- GGTGCGGGT**TTC**CATGCGTGGAAGGCGGGT -3’
Y516A	5’- TTTGGTGCGGGT**GCC**CATGCGTGGAAG -3’
